# Development and characterization of a high-throughput in vitro cord formation model insensitive to VEGF inhibition

**DOI:** 10.1186/1756-8722-6-31

**Published:** 2013-04-27

**Authors:** Beverly L Falcon, Belinda O’Clair, Don McClure, Glenn F Evans, Julie Stewart, Michelle L Swearingen, Yuefeng Chen, Kevin Allard, Linda N Lee, Kuldeep Neote, Dyke P McEwen, Mark T Uhlik, Sudhakar Chintharlapalli

**Affiliations:** 1 Eli Lilly and Company, Department of Cancer Angiogenesis, Lilly Corporate Center, Indianapolis, IN, 46285, USA; 2 Eli Lilly and Company, Department of BioTDR, Lilly Corporate Center, Indianapolis, IN, 46285, USA; 3 Eli Lilly and Company, Department of Tailored Therapeutics, Lilly Corporate Center, Indianapolis, IN, 46285, USA; 4 Essen BioScience, Inc., 300 West Morgan Road, Ann Arbor, MI, 48108, USA

**Keywords:** Angiogenesis, Vascular endothelial growth factor (VEGF), Adipose derived stem cells (ADSC), Endothelial colony forming cells (ECFC)

## Abstract

**Background:**

Anti-VEGF therapy reduces tumor blood vessels, however, some vessels always remain. These VEGF insensitive vessels may help support continued tumor growth and metastases. Many *in vitro* assays examining multiple steps of the angiogenic process have been described, but the majority of these assays are sensitive to VEGF inhibition. There has been little focus on the development of high-throughput, *in vitro* assays to model the vessels that are insensitive to VEGF inhibition.

**Methods:**

Here, we describe a fixed end-point and kinetic, high-throughput stem cell co-culture model of cord formation.

**Results:**

In this system, cords develop within 24 hours, at which point they begin to lose sensitivity to VEGF inhibitors, bevacizumab, and ramucirumab. Consistent with the hypothesis that other angiogenic factors maintain VEGF-independent vessels, pharmacologic intervention with a broad spectrum anti-angiogenic antagonist (suramin), a vascular disrupting agent (combretastatin), or a combination of VEGF and Notch pathway inhibitors reduced the established networks. In addition, we used our *in vitro* approach to develop an *in vivo* co-implant vasculogenesis model that connects with the endogenous vasculature to form functional blood vessels. Similar to the *in vitro* system, over time these vessels become insensitive to VEGF inhibition.

**Conclusion:**

Together, these models may be used to identify novel drugs targeting tumor vessels that are not sensitive to VEGF inhibition.

## Background

Preclinical studies indicate that most solid tumors require angiogenesis, the formation of new blood vessels from existing vessels, for growth, survival, and metastasis. While many factors regulate tumor angiogenesis, vascular endothelial growth factor (VEGF) appears to have a dominant role, inducing vascular permeability, endothelial cell proliferation and migration, and new blood vessel growth. Numerous drugs have been developed to target the VEGF pathway with receptor tyrosine kinase inhibitors, with soluble decoy receptors, or with antibodies targeting the VEGF ligand or receptor. Inhibition of VEGF signaling reduces tumor growth in many preclinical models [1,2], however, the benefits of targeting VEGF in mouse models have not completely translated to the clinic. While the FDA has approved multiple VEGF pathway inhibitors for clinical treatment of certain cancers, not all patients benefit from these treatments. Some tumors may initially respond but eventually become refractory, while others show no clinical benefit of inhibiting the VEGF pathway [3]. Some preclinical models have even shown resistance and increased metastatic spread associated with VEGF inhibition [4-6].

Both preclinical and clinical studies have shown that despite significant reductions in tumor blood vessels with VEGF signaling blockade, some tumor blood vessels remain [7,8]. The blood vessels that remain have a distinct phenotype typically associated with more pericyte coverage [8-13]. There are a number of possible explanations for this effect. First, the initial reduction in tumor blood vessels leads to tumor cell hypoxia, which, in turn, can cause tumor cells to either secrete more VEGF to overcome the anti-VEGF therapy or stimulate the release of other pro-angiogenic cytokines [4,14-16]. “Vascular normalization” may also play a role in VEGF resistant tumor vessels. VEGF inhibitors can transiently improve pericyte and basement membrane coverage, decrease tumor vessel tortuosity and hyperpermeability, and increase oxygen and drug delivery [10,17,18]. These vessels may be formed via normalization of the atypical phenotype associated with tumor vessels or a pruning of the abnormal vessels leaving behind pre-existing vessels that have a more normal phenotype. Studies by Hal Dvorak’s group indicate that tumor blood vessels are heterogeneous consisting of at least six distinctly different blood vessel types: (1) “mother” vessels, (2) glomeruloid microvascular proliferations, (3) vascular malformations, (4) capillaries, (5) feeder arteries (6) and draining veins [19,20]. Interestingly, only subpopulations of these vessels are sensitive to VEGF inhibition. Immunodeficient mice expressing VEGF-A_164_ initially form vessel subtypes such as “mother” vessels and GMPs that are sensitive to VEGF inhibitors while later stage vessels are VEGF-independent [20,21]. Thus, the developmental stage of tumor vasculature is critical to anti-VEGF therapy sensitivity and the lack of good *in vitro* resistance models has slowed the development of non-VEGF anti-angiogenic therapies. In particular, studies should be developed to identify novel ways of targeting the tumor blood vessels that remain or are insensitive to VEGF inhibition.

Many *in vitro* assays have been developed that examine multiple steps in the angiogenic process. These assays interrogate sprouting and tip formation, migration and proliferation, lumen formation, and tube or cord formation. *In vivo* assays also look at many of these similar processes. The majority of these assays, however, are driven by the addition of VEGF or other growth factors to the system and remain sensitive to VEGF inhibition [22-25]. Disrupting established vessels, cords, or tubes which may be insensitive to VEGF inhibitors, however, has not been a major focus of *in vitro* or *in vivo* approaches. Here, we describe an *in vitro* cord formation assay that demonstrates insensitivity to VEGF inhibition. Similar to what is seen *in vivo*, resistance to VEGF inhibition is associated with cord maturity and pericyte association. The advantage of this approach is its increased throughput and ability to identify novel anti-angiogenic agents that can inhibit VEGF-independent vessels. Finally, we show the translatability of this *in vitro* approach using an *in vivo* model of vasculogenesis to validate the effectiveness of novel treatments on the ability to decrease blood vessels that are insensitive to VEGF inhibition.

## Results

### Characterization of multiple *in vitro* angiogenesis models

**Figure 1:**
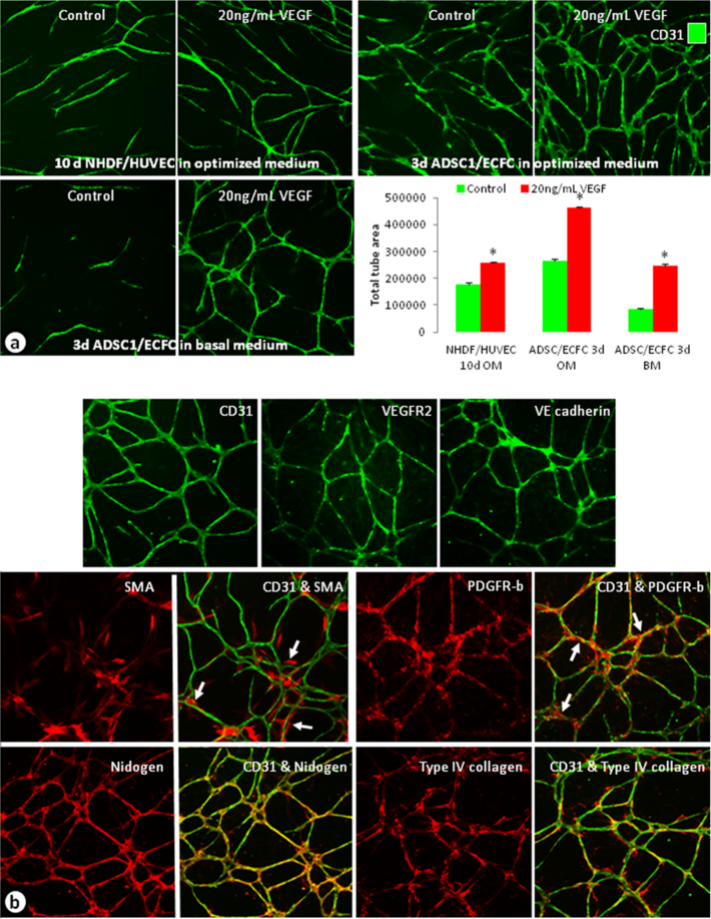
**Characterization of co-cultured cord formation assays.** (**a**) Unstimulated or VEGF-stimulated (20 ng/mL) cords stained with CD31 from co-cultures of NHDFs and HUVECs (top left), ADSCs and ECFCs in optimized medium (top right), and ADSCs and ECFCs grown in basal medium (bottom left). Graph compared the total tube areas of the cords from the different assay systems. n = 3–5 per group. * = p < 0.0001 vs. respective control. (**b**) Images of 5d ADSC and ECFC cords grown in basal medium and stimulated with 20 ng/mL VEGF. Endothelial cells were labeled with CD31, VEGFR-2, or VE-cadherin (top), mural cells or pericytes were labeled with SMA or PDGFR-β (middle), and vascular basement membrane was identified by nidogen and type IV collagen antibodies (bottom). Arrows indicate areas where pericytes labeled with SMA or PDGFR-β were associated with the cords.

Multiple *in vitro* models of angiogenesis or cord formation were examined (Figure 1). Traditionally, co-cultures of HUVECs and NHDFs have been used to analyze and quantify growth factor and drug effects on angiogenesis [26]. Recently, a co-culture model of ECFCs and ADSCs, which has a shorter experimental duration and presence of pericyte biology, has been described [22]. In all of the models examined, cord formation occurred in the controls with increased cord formation induced by 20 ng/mL VEGF (Figure 1a). We observed a 44% increase in cords in the NHDF/HUVEC co-culture model while there was a 76% increase in cords in the ADSC/ECFC co-culture model at this VEGF concentration (Figure 1a). The optimized media used for these assays, however, contain serum and angiogenesis related growth factors such as epidermal growth factor (EGF) and basic fibroblast growth factor (FGF). In order to reduce background cord formation and increase responsiveness to exogenously added angiogenic growth factors, a basal media (BM) was developed which lacks serum and any additional growth factors. When the ADSC/ECFC co-culture was run in BM, the background cord formation decreased by 68% and there was a 194% increase in cord formation with the addition of VEGF (Figure 1a). Immunocytochemical characterization showed that cords formed in the ADSC/ECFC co-cultures express multiple markers common to the *in vivo* vasculature [27-29] (Figure 1b). CD31 (PECAM-1), VEGFR-2, and VE-cadherin were expressed by the endothelial cells forming the cords (Figure 1b). In addition, only ADSCs that were in close proximity with endothelial cells differentiated into cells expressing SMA and PDGFR-β, indicative of a pericyte-like phenotype [28] (Figure 1b, arrows). These pericyte markers were not expressed in the ADSC feeder layer found away from the cords. Finally, vascular basement membrane markers, such as nidogen and type IV collagen, were expressed and associated with the cords in this co-culture system (Figure 1b). In contrast, in the NHDF/HUVEC co-culture model, the cords expressed endothelial and basement membrane markers, but pericyte markers were not expressed (data not shown).

### Time course of ADSC/ECFC cord formation

**Figure 2:**
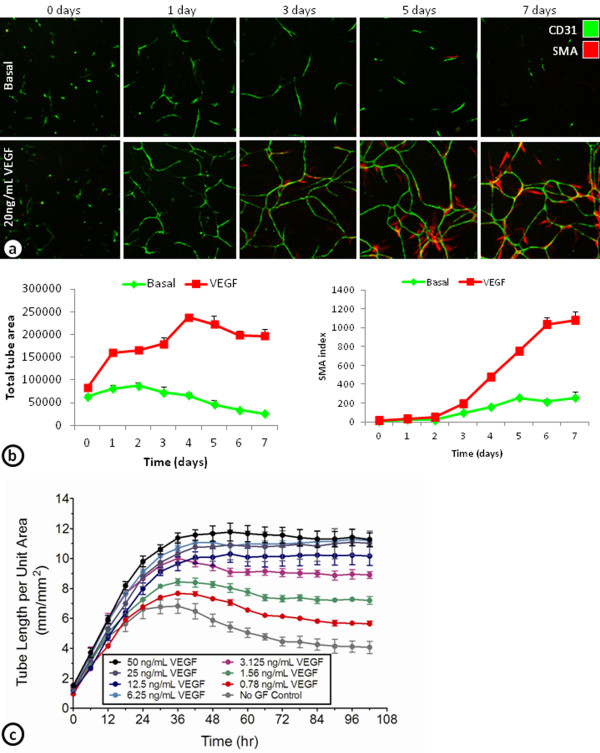
**Time course of cord formation in ADSC and ECFC co-cultures grown in basal medium.** (**a**) Images of endothelial cells stained with CD31 and SMA-positive pericytes in the ADSC/ECFC co-culture assay system either unstimulated (basal; top) or stimulated with 20 ng/mL VEGF (bottom) at 0, 1, 3, 5, and 7 days. (**b**) Graphs of a time course from 0–7 days showing total tube area of the cords (left) and its associated SMA index (right). (**c**) Continuous monitoring of the tube length per unit area of GFP labeled ECFCs in the ADSC/ECFC co-culture assay system in basal media plus with no stimulation (gray) or a concentration response of VEGF from 0 to 102 hours.

To further characterize the development of basal and VEGF-induced cords and its associated SMA cells, ADSC/ECFC co-cultures were examined from 0–7 days (Figure 2a and b). After 1 day, many of the basal and VEGF-induced cords have already formed. The basal cords were only stable for a few days, before reductions in total tube area were seen beginning around day 3. VEGF-induced cords were stable over the 7 day time course with slight increases in total tube area after the first 24 hours (Figure 2a and b). The increase in SMA index was not observed until day 3, and increased dramatically over the next 48 hours (Figure 2a and b). These results indicate that VEGF-induced cords are initially formed within the first day. After the first day, however, the cords appear to remodel and may become more stable by the differentiation of the ADSCs into SMA expressing pericyte-like cells.

Continuous monitoring of cord formation using GFP-expressing ECFCs cultured with ADSCs demonstrated concentration dependent increases in VEGF induced cord formation (Figure 2c). Similar to the fixed endpoint studies, the increased VEGF induced cord formation was found in the first 24–36 hours. After 36 hours, the higher concentrations of VEGF-induced cords were stable over the next 3 days (Figure 2c).

### Targeting the components of the cord formation system

**Figure 3:**
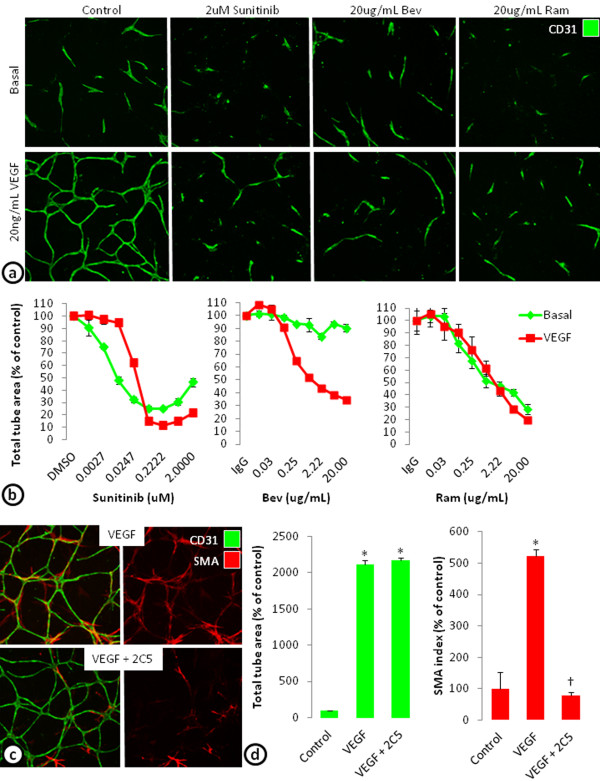
**Targeting cord formation or pericyte association in the ADSC/ECFC co-culture model.** (**a**) CD31 stained basal or VEGF stimulated cords at 3 days treated with 2 μM Sunitinib, 20 μg/mL bevacizumab (Bev), or 20 μg/mL ramucirumab (Ram). (**b**) Graphs of a concentration response of sunitinib, bevacizumab (Bev), and ramucirumab (Ram) on total tube area of basal and VEGF stimulated cord formation after 3 days. (**c**) VEGF stimulated cords were established for 4 days then treated with control or a PDGFR-β inhibitor, IMC-2C5 (2C5) for 3 days. Images show the effects of control and IMC-2C5 on VEGF stimulated endothelial cords stained with CD31 (green) and SMA-positive pericytes (red). (**d**) Graphs of total tube area (left) and SMA index (right) of VEGF stimulated cords with or without PDGFR-β inhibition. n = 3 per group. * = p < 0.001 vs. Control. † = p < 0.01 vs. VEGF.

To determine whether VEGF-induced cords can be targeted with anti-VEGF therapy, cords were treated with a receptor tyrosine kinase inhibitor targeting the VEGF receptors among others (sunitinib), an antibody targeting the VEGF-A ligand (avastin; bevacizumab), or an antibody targeting VEGFR-2 (IMC-1121B; ramucirumab) (Figure 3a). Blocking VEGF signaling with sunitinib, bevacizumab, or ramucirumab all concentration-dependently reduced VEGF-driven cord formation in the ADSC/ECFC co-culture system. Sunitinib (0.2 μM) maximally decreased VEGF-induced total tube area by 89% (EC_50_ = 0.027 μM), bevacizumab (20 μg/mL) by 65% (EC_50_ = 0.174 μM), and ramucirumab (20 μg/mL) by 80% (EC_50_ = 0.623 μM) (Figure 3a and b). In addition to VEGF-induced cords, sunitinib and ramucirumab decreased basal total tube area by 75% and 72% respectively, while bevacizumab only decreased basal cords by 10% (Figure 3a and b). While the inhibitors of VEGF signaling decreased cord formation in a concentration-dependent manner, even at the highest concentrations, cords were not completely eliminated. This may indicate that the remaining cords are dependent on other growth factors secreted by the feeder layer of ADSCs. In fact, examination of cords indicate that the basal cords formed in the ADSC/ECFC co-culture system are also dependent on HGF [22] (Additional file 1: Figure S1).

The ability to target SMA positive pericyte differentiation was also examined. Previous studies indicate that PDGF expression and stimulation of its receptor, PDGFR-β, are important for pericyte recruitment [30-32]. Here, cords were allowed to form for 4 days to allow for some pericyte differentiation. After 4 days, the cords were treated with 20 μg/mL IgG_1_ or a PDGFR-β blocking antibody (IMC-2C5; [33]). Inhibition of PDGFR-β signaling with IMC-2C5 decreased the SMA index induced by VEGF by 79%, indicating a reduction in pericyte coverage, but the total tube area was not affected (Figure 3c and b).

### Development of VEGF insensitive cords

**Figure 4:**
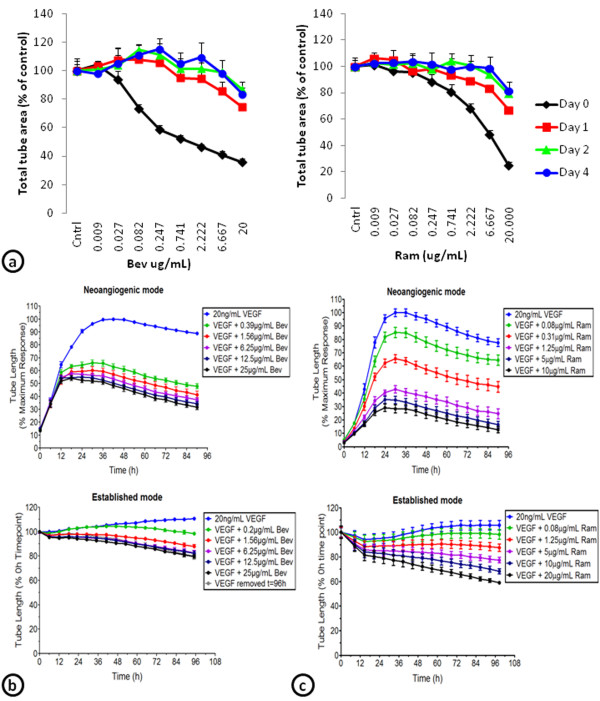
**VEGF stimulated cords become insensitive to inhibitors of VEGF signaling in the ADSC/ECFC co-culture model.** (**a**) Cords were established for 0, 1, 2, or 4 days, then treated with a concentration response of bevacizumab (Bev; left) or ramucirumab (Ram; right). Total tube areas for each were graphed. (**b**) Continuous monitoring of the effect of a concentration response of bevacizumab (Bev) on cord tube length beginning on day 0 (neoangiogenic mode; top) or after 4 days of establishment (established mode; bottom). (**c**) Continuous monitoring of the effect of a concentration response of ramucirumab (Ram) on cord tube length beginning on day 0 (neoangiogenic mode; top) or after 4 days of establishment (established mode; bottom).

To determine whether cords remain sensitive to inhibitors of VEGF signaling, VEGF-induced cords were allowed to establish for 0, 1, 2, or 4 days prior to addition of bevacizumab or ramucirumab. Bevacizumab (20 μg/mL) decreased total tube area by 64% when given on day 0, by 25% on day 1, 13% on day 2, and 16% on day 4 (Figure 4a). Cord formation was reduced by 75% when ramucirumab (20 μg/mL) was given on day 0, 33% on day 1, 21% on day 2, and 18% on day 4 (Figure 4a). These results indicate that once established, cords become increasingly resistant to VEGF inhibition. To further characterize this insensitivity to VEGF inhibition, continuous live-cell monitoring of the cords after ramucirumab or bevacizumab treatment at day 0 (neoangiogenic mode) or day 4 (established mode) was compared (Figure 4b and c). Bevacizumab (25 μg/mL) decreased neoangiogenic cord formation by 70%, but only reduced established cord formation by 20% (Figure 4b). Likewise, ramucirumab treatment (10 μg/mL) decreased neoangiogenic cord formation by 90% and only decreased established cord formation by 30% (Figure 4c).

### *In vivo* model of VEGF insensitive vessels

**Figure 5:**
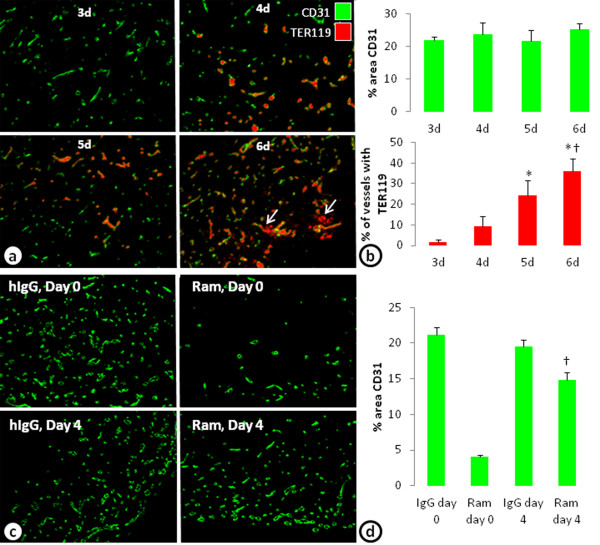
***In vivo *model of vasculogenesis develops VEGF-independent cords.** (**a**) An *in vivo* model of vasculogenesis in which ADSCs and ECFCs co-implanted into the flank develop blood vessel like structures stained with CD31. The vessels anastamose with the host vessels and have blood cells (erythrocytes; TER119) associated with the vessels beginning on day 4. At later times, however, hemorrhage indicated by TER119 staining not associated with the blood vessels were seen (arrows). (**b**) Graph of the percent area of CD31 and the percent of vessels associated with erythrocytes (TER119) after 3–6 days of growth in the flank of a mouse. n = 8 per group. * = p < 0.05 vs. day 3. † = p < 0.01 vs. day 4. (**c**) Images of blood vessels stained with CD31 within the implants after treatment with IgG or ramucirumab (Ram) beginning on day 0 (top) or after 4 days (bottom) of establishment. (**d**) Graph of the percent area of CD31 after 4 days of treatment with IgG or ramucirumab beginning on day 0 or day 4. n = 10 per treatment group. * = p < 0.0001 vs. all other treatment groups. † = p < 0.01 vs. all other groups.

A limitation of any *in vitro* model is its translatability to *in vivo* biology. Here, to investigate whether similar VEGF-independent vessels can be established *in vivo*, a co-implant model of *in vivo* vasculogenesis with ADSCs and ECFCs was performed. Injection of a mixture of ADSCs and ECFCs in Matrigel develop blood vessels within 3 days. Evidence of blood perfusion (identified with an erythrocyte marker, TER119) in the vessels was seen beginning at 4 days and increased with additional time (Figure 5a and b). At 6 days, however, evidence of hemorrhage, or TER119 not associated with blood vessels became evident (Figure 5a, arrows). Four-day treatment with ramucirumab beginning on day 0 decreased the percent area of CD31 by 81%. However, when ramucirumab treatment was given for 4 days beginning on day 4, the percent area of CD31 was only reduced by 24% (Figure 5c and d); recapitulating the *in vitro* observations.

### Targeting VEGF-independent cords

**Figure 6:**
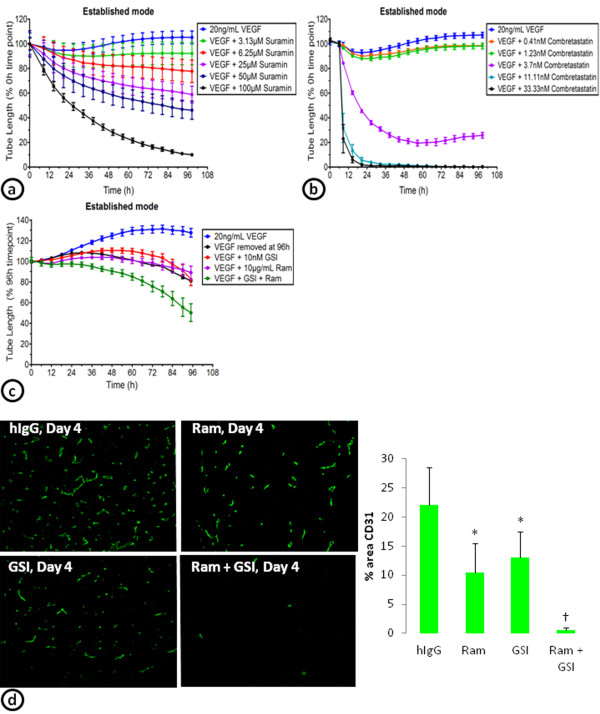
**Targeting the VEGF-independent cords.** VEGF stimulated ADSC/ECFC co-cultures established for 4 days were treated with a concentration response of suramin (**a**) or combretastatin (**b**). The effects on the tube length of the established cords were continuously monitored from the day of drug addition. **(c)** Continuous monitoring of the effect of ramucirumab (Ram), a gamma secretase inhibitor (GSI; LY411575), or the Ram/GSI combination on the tube length of 4 day established cords. (**d**) Images of blood vessels stained with CD31 within the implants after treatment with IgG, ramucirumab (Ram), a gamma secretase inhibitor (GSI; LY411575), or the combination of Ram and the GSI beginning after 4 days of establishment (left). Graph of the percent area of CD31 after 4 days of treatment beginning on day 4 (right). n = 10 per treatment group. * = p < 0.01 vs. hIgG control. † = p < 0.001 vs. all other groups.

A number of different mechanisms, including the upregulation of other angiogenic pathways such as the Notch pathway, have been described to play a role in the development of tumor vessels that are insensitive to anti-VEGF therapy [4,14,15,34]. To determine whether other classes of anti-vascular therapy can reduce cords that are insensitive to VEGF inhibition, a broad spectrum anti-angiogenic antagonist (suramin), a vascular disrupting agent (combretastatin), and a combination therapy of a VEGFR-2 inhibitor (ramucirumab) and a gamma secretase inhibitor (GSI; LY411575 [35,36]) were tested on established cords (Figure 6). VEGF-induced cords were allowed to establish for 4-days prior to addition of suramin, combretastatin, or the ramucirumab/GSI (LY411575) combination. Suramin treatment (100 μM) decreased VEGF-established cords by 90% (Figure 6a), combretastatin (11 nM) decreased cords by 100% (Figure 6b), and the combination of ramucirumab (10 μg/mL) and a GSI (LY411575; 10 nM) decreased established cords by 50% (Figure 6c). The reduction in established cords with the combination of ramucirumab and a GSI (LY411575) was greater than either of the drugs alone (Figure 6c). Similarly, in the co-implant model of *in vivo* vasculogenesis, ramucirumab or the GSI (LY411575) alone led to slight reductions in vessels that were allowed to establish for 4 days prior to treatment. However, the combination of ramucirumab and the GSI (LY411575) almost completely eliminated the vessels (Figure 6d).

## Discussion

Results from this study indicate that a co-culture system of progenitor cells and endothelial cells can create a cord network with components found in the vasculature: endothelial cells, pericytes, and basement membrane. Exogenously added VEGF can stimulate cord formation and the cords that develop become insensitive to VEGF inhibition as the cords mature. An *in vivo* co-implant model of vasculogenesis, using the same progenitor and endothelial cells as the *in vitro* approach, develops functional blood vessels that anastamose with the host vasculature. These vessels also become insensitive to VEGF pathway inhibition with time. Together, these studies indicate that the combined use of an *in vitro* high-throughput established cord formation assay and an established *in vivo* co-implant model of vasculogenesis can be used to identify novel drugs that can target VEGF-independent blood vessels.

Here, we investigate two different *in vitro* models of angiogenesis. The first model uses co-cultures of human umbilical vein endothelial cells (HUVEC) with normal human dermal fibroblasts (NHDF) and the other uses co-cultures of endothelial colony forming cells (ECFC) with adipose-derived stem cells (ADSC). When seeded with NHDF, the HUVECs recapitulate the major phases of the angiogenic process, initially proliferating and migrating into endothelial clusters followed by differentiation and branching into complex networks over the 7–10 day assay. Under basal conditions, little tube formation occurs, while VEGF addition on day 3–4 stimulates tube formation in a concentration-dependent manner [37-39]. Further characterization of the HUVEC/NHDF approach shows that the pharmacological and physiological effects on endothelial cell biology are highly translatable to previous *in vivo* characterizations, exemplified by DLL4/Notch inhibition resulting in increased branch point formation late in the angiogenic process [39-41]. One of the major advantages of the ADSC and ECFC co-culture model is that the process of developing cords occurs quickly and incorporates a pericyte-like biology associated with the cords. Unlike the NHDF/HUVEC model and other similar models, in the ADSC/ECFC model, the majority of VEGF-driven cords are formed within the first 24 hours [23,24,38,42]. Additional stimulation allowed for further remodeling of the vessels and differentiation of the ADSCs into SMA or PDGFR-β expressing pericyte-like cells. Characterization of these SMA associated cords indicates that drugs targeting VEGF or PDGF can inhibit the cords or the pericytes, respectively, similar to what has previously been shown *in vivo*[8,28]. Further, VEGF is not the only growth factor that induces rapid cord formation. Other pro-angiogenic growth factors, including FGF and EGF, also induce cord formation in a concentration-dependent manner and exhibit different phenotypes and kinetics (manuscript under preparation). In addition to the assay duration and the cord similarities to *in vivo* vascular structure, the use of basal media in the ADSC/ECFC co-culture assay has dramatically increased the response window for drug screening purposes.

Interestingly, we found that inhibition of the VEGF receptor with sunitinib or ramucirumab leads to decreases in basal and VEGF driven cord formation, but inhibition of the ligand with bevacizumab only affected the VEGF driven cords. There may be several explanations for this effect. Endothelial cells can make VEGF and signal in an autocrine fashion. In fact, previous studies using endothelial cell specific knockout of VEGF indicates that autocrine VEGF signaling is required for the homeostasis of blood vessels [43]. It is also feasible that ligand-independent mechanisms or signaling through heterodimerization of VEGFR2 with other receptors may play an important role in basal cord formation [44]. Internalization of the receptor with ramucirumab or the multi-targeted nature of Sunitinib may play a role as well. Finally, VEGF secreted in the co-culture system may get bound to the extracellular matrix, where it may not be accessible to VEGF antibodies, but may still be able to be affected by receptor inhibition. These possibilities require further exploration as it is unclear what mechanism or mechanisms are involved in the ECFC/ADSC co-culture assay.

Unlike most tube and cord formation assays, established cords in the ADSC and ECFC co-culture system lose their dependence on VEGF once the cords are developed. Even after 1 day of establishment, the cords are less sensitive to multiple inhibitors of the VEGF signaling pathway. The mechanism of this VEGF-independence is not clear. Pericyte coverage is thought to make vessels insensitive to VEGF inhibition, but in this assay, we see increased VEGF independence after 1 day even though the SMA differentiation does not occur until day 3. In addition, inhibition of the PDGFR after 4 days of establishment decreased the SMA index but did not significantly alter total tube area. While it is still possible that pericytes play an important role in maintenance of established vessels, in this assay system other growth factors secreted by the ADSC feeder layer likely play a major role in maintaining the cords once they have been created. Clearly, there are some cords that can form without the addition of VEGF. Previous studies and our data indicate that HGF is highly expressed by the ADSCs and contributes to basal cord formation [22]. *In vivo* studies show that inhibition of VEGF and the HGF receptor, c-Met, decrease tumor vessels more than VEGF inhibition alone [29]. Together, these results indicate that the HGF secreted by the feeder layer may have an important role in maintenance of the established cords. In addition, we show that suramin, a broad-spectrum antagonist that inhibits various angiogenesis-related growth factors such as insulin-like growth factor, epidermal growth factor, platelet-derived growth factor, VEGF, and basic fibroblast growth factor, is able to reduce the VEGF-independent cords. Together, these results indicate that other growth factors secreted from the ECFCs or ADSC feeder layer may maintain cords following the initial VEGF stimulation.

Using an *in vivo* co-implant model of vasculogenesis with ADSCs and ECFCs, functional vessels can form after anastamosing with the host vasculature. These vessels form over 3 days and blood cells labeled with TER119 can be seen beginning 4 days after the cells are injected into the flank. Treatment of the vasculogenic plugs with a VEGF inhibitor dramatically decreases blood vessel formation if given at the beginning of the assay. If, however, VEGFR signaling was not inhibited until the vessels have established and have blood flow (at day 4), there is little effect. While the mechanism of this insensitivity is not know, it would be interesting to characterize our VEGF independent vessels to determine whether they have similar phenotypes as those described by the Dvorak laboratory [20,21]. Nonetheless, these results are consistent with our high-throughput *in vitro* assay and provide a unique *in vivo* model to examine the effects of novel drugs on vessels that are insensitive to VEGF inhibition.

As proof of principle, a broad-spectrum anti-angiogenic inhibitor (suramin), a vascular disrupting agent (combretastatin), and a combination of a gamma secretase and VEGF inhibitor were tested on VEGF established cords. Suramin blocks a variety of growth factors including many angiogenesis-related factors. One of the proposed mechanisms of VEGF-independent tumor vessels is that inhibition of VEGF leads to induction of other proangiogenic factors [4,14,15,34]. Suramin is likely able to block many of these other proangiogenic factors to reduce the cords in our established cord assay system.

The vascular disrupting agent combretastatin is a microtubule-depolymerizing agent which binds to tubulin dimers to prevent microtubule polymerization. This results in mitotic arrest and apoptosis of endothelial cells. In addition, combretastatin disrupts the endothelial cell junction molecule (VE-cadherin) leading to vascular collapse [45] and *in vivo* studies show that combretastatin is able to reduce immature vessels [45,46]. We observed reductions in established cords with combretastatin treatment. Clearly, while combretastatin may not reduce all mature vessels *in vivo*, it is able to target a unique population of vessels or cords that are insensitive to VEGF inhibition. In fact, preclinical and clinical studies indicate that combining combretastatin with bevacizumab is more efficacious than either inhibitor alone [47,48].

A recent *in vivo* study indicates that VEGF-independent vessels are driven by DLL4-Notch signaling and are sensitive to gamma secretase inhibition [34]. Consistent with this novel strategy to overcome anti-angiogenic resistance, a gamma secretase inhibitor was tested in our *in vitro* and *in vivo* models alone or in combination with inhibition of VEGF signaling. In the *in vitro* system, treatment with either compound alone prevented a slight increase in cords associated with feeding the cells with fresh VEGF, but did not disrupt established networks. However, when VEGF and gamma secretase inhibitors were combined, there was a reduction in the number of cords. Similarly, in the in vivo co-implant model, ramucirumab or the gamma secretase inhibitor alone elicited a slight reduction in the vessels, but the combination reduced the vessels significantly more. These results indicate that our established cord assays may be used to identify new pathways involved in anti-VEGF/VEGFR directed therapy resistance and potential combinatorial strategies.

Many current angiogenesis assays used to screen anti-angiogenic agents are highly VEGF dependent. However, from preclinical and clinical analysis, there clearly exists a population of tumor vessels that are insensitive to VEGF inhibition. Thus, angiogenic assays are needed in which novel agents can be tested for their effectiveness on vessels which are not dependent on VEGF. The ECFC/ADSC assay is high throughput and relatively quick. Results can be obtained in approximately a week and can be run in 96-well and 384-well formats and similar co-culture approaches have previously been used in high-throughput drug discovery [24,49]. In addition, labeling the ECFCs with GFP is a feasible approach to monitor cord formation and effects on established cords using continuous live-cell monitoring. Together, these results indicate that a co-culture cord formation system with ADSCs and ECFCs is a useful method to identify and characterize novel drugs on VEGF-independent cords. It would be interesting to identify selective markers on tumor vessels that remain after VEGF therapy and determine if the same markers exist in this co-culture system. If so, these in vitro and in vivo systems would be conducive to interrogate the mechanisms by which vessels become insensitive to VEGF inhibition though use of shRNA/siRNA knockdowns. With more and more studies being published regarding mechanisms of VEGF resistance, additional targets should be tested in this *in vitro* co-culture system.

## Conclusions

Despite in vivo evidence that VEGF independent vessels exist, the majority of the in vitro assays used are dependent on VEGF. We described an in vitro cord formation assay that shows insensitivities to inhibition of the VEGF pathway. In addition, we were able to show the translatability of this assay using an in vivo model of vasculogenesis. Together, the combined use of this *in vitro* high-throughput established cord formation assay and an established *in vivo* co-implant model of vasculogenesis can be used to identify novel drugs that can target VEGF-independent blood vessels.

## Methods

### Cell lines and media

Human adipose derived stem cells (ADSCs) isolated from lipoaspirates collected during surgical liposuction procedures were purchased from Lonza (Allendale, NJ). Cells were grown in EGM2-MV media (Cambrex; Walkersville, MD) and used at passage 4–6. Endothelial colony forming cells (ECFCs) isolated from cord-blood derived endothelial cells were grown on Collagen I coated flasks in EGM2-MV media supplemented with an additional 5% FBS and used at passage 7–10 (Lonza). For studies examining cord formation over time with continuous live-cell monitoring, ECFCs were lentivirally transduced to express CytoLight Green, a soluble variant of GFP, and optimized for imaging in the IncuCyte™ imaging system. Human umbilical vein endothelial cells (HUVECs) and normal human dermal fibroblast (NHDF) cells and media were purchased from Cambrex. HUVECs were grown in EGM media with 10% FBS and NHDF cells were maintained in EGM-2 media.

### Co-culture assay of endothelial cells and fibroblasts

HUVEC and NHDF co-culture cord formation assays were performed with AngioKit optimized media (TCS Cellworks, Birmingham, UK) as previously described [26,37-39]. Briefly, 20K NHDF cells in 100 μL of media were plated in each well of a 96-well plate and incubated overnight at 37°C, 5% CO_2._ The next day, HUVECs were added on top of the NHDF cells at 1800 cells/well in 100 μL and incubated overnight at 37°C, 5% CO_2._ On the third day and every subsequent third day, the media was changed to optimized media containing 20 ng/mL VEGF (R&D Systems). On day 10, the co-culture was fixed, stained, and imaged as described below.

### Neoangiogenic ADSC and ECFC co-culture cord formation assay

ADSC and ECFC co-culture assays were performed with AngioKit optimized media, basal media (MCDB-131 medium with 30 μg/mL L-ascorbic acid 2-phosphate, 1 μM dexamethasone, 50 μg/mL tobramycin, 10 μg/mL r-transferrin AF, and 10 μg/mL insulin) or basal media plus (MCDB-131 medium with 0.3% FBS, 30 μg/mL L-ascorbic acid 2-phosphate, 50 μg/mL tobramycin, 10 μg/mL r-transferrin AF, and 10 μg/mL insulin). ADSCs were plated in 96-well plates at 40–50K cells per well in 100 μL and incubated overnight at 37°C, 5% CO_2._ The next day, the media was removed and 4–5K ECFCs per well in 50–100 μL of media was plated on top of the ADSC monolayer and incubated at 37°C, 5% CO_2_ for 3–6 hours before the addition of growth factors and inhibitors. After the ECFCs attach, growth factors and test agents were added to the 50–100 μL of media at 2–5× to achieve the final concentrations as indicated. Co-cultures were grown for 0–7 days at which time the cells were fixed, stained, and imaged as described below.

### Established ADSC and ECFC co-culture cord formation assay

Established ADSC and ECFC co-culture assays were plated as described above for the neoangiogenesis assay. After the ECFCs were allowed to attach, 20 ng/mL VEGF was use to stimulate and establish the cord network. After 1–4 days the media was changed to contain fresh VEGF in the presence or absence of inhibitors at the indicated concentrations. After addition of the inhibitors, cultures were allowed to grow an additional 3–4 days before the cells were fixed, stained, and imaged as described below to investigate network disruption or cord regression.

### Fixation and staining of fixed endpoint cords

At the completion of the assay, ADSC/ECFC cords were fixed and permeabilized with either 70% ice cold ethanol for 20–30 minutes or 3% paraformaldehyde for 10 minutes followed by 70% ice cold ethanol for 20 minutes. Cells were blocked with PBS + 1% bovine serum albumin (BSA) for 30 minutes at room temperature. Primary antibodies were diluted in PBS + 1% BSA and stained either sequentially or in combination for >90 minutes at 37°C. Endothelial cells were identified with sheep anti-CD31 (PECAM-1; Sigma; 1:200), rabbit anti-VEGFR-2 (55B11; Cell Signaling; 1:50), or goat anti-VE-cadherin (Santa Cruz; 1:50) antibodies. Cy3 conjugated mouse anti-smooth muscle actin (SMA; Sigma; 1:200) and rabbit anti-platelet-derived growth factor receptor beta (PDGFR-β; Y92; LifeSpan; 1:50) antibodies identified pericytes associated with the cords. Vascular basement membranes were identified with goat anti-Nidogen (R&D Systems; 1:50) and goat anti-type IV collagen (Millipore; 1:50) antibodies. After a brief wash, secondary AlexaFluor 488- and 555-conjugated donkey anti-sheep, donkey anti-rabbit, donkey anti-goat secondary antibodies (Invitrogen; 1:400) were incubated for ~60 minutes at room temperature. Nuclei were identified with Hoechst 33342 (Invitrogen; 1:1000) for 5 minutes at room temperature. After Hoechst staining, the cells were washed and imaged as described below.

### Fixed endpoint imaging and quantification

Cord formation images were captured using a Cellomics Arrayscan VTI and analyzed with the Tube Formation bio-application reading at a magnification of 5×. Objects were identified using an algorithm to detect CD31 staining of cords. Total tube area was calculated from 9 fields for each well with 3–4 wells for each treatment. SMA index was calculated from the intensity of the SMA staining and related to the number of cords/tubes.

### Continuous monitoring of cord formation

ADSCs and ECFCs transduced with CytoLight Green were seeded for the assay as described above. After 3–4 hours at 37°C, the cells were treated with test reagents (growth factors ± compounds or antibodies), placed into the IncuCyte FLR for imaging, and allowed to form networks over the course of the 4 day experiment. If running the assay in neoangiogenic mode, looking at the inhibition of tube formation, the assay was terminated at the 96 hour time point. If studying tube regression was desired, the assay was run in established mode. To do this, growth factor-driven networks were formed over the first 96 hours of the assay. At this point, a full media replacement occurred including fresh growth factor in the presence or absence of test agent. The assay plate was then placed back in the IncuCyte FLR and imaged over the desired time frame to quantify regression of established networks.

For imaging and quantification, phase-contrast and fluorescent images were automatically collected every 6 hours in the IncuCyte FLR to detect network formation using the Tiled Field of View (FOV) mosaic imaging mode. The integrated Angiogenesis Analysis Module was used to identify the fluorescent signal from background in order to quantify multiple assay metrics, such as tube length and branch formation, for each time point. In the first step of the process, the angiogenesis algorithm analyzed each fluorescent image and assigned a segmentation mask that closely resembles the *in vitr*o network. From here, the mask was refined and filtered to exclude non-tube forming events, specifically measuring angiogenesis over time. Kinetic plots of the angiogenesis metrics was generated using the IncuCyte software, allowing for a direct comparison of test agent treatments to validated control conditions (Figure 2b).

### *In vivo* vasculogenesis assay

ADSCs and ECFCs were mixed in a ratio of 1:4 (0.5 × 10^6^/2 × 10^6^ cells/mL) in Matrigel (BD Biosciences) and injected (0.2 mL/implant) subcutaneously into the flank of female athymic nude mice as previously described [50,51]. Three to six days following implantation, implants were collected and placed into zinc-tris fixative. Treatments with IgG or IMC-1121B (ramucirumab; 10 mg/kg, ip) began on day 0 or day 4. Treatments with the gamma secretase inhibitor (GSI, LY411575; 3 mg/kg QD, ip) alone or in combination with ramucirumab began on day 4. The concentration of drugs used was determined from dose response studies (data not shown). Implants were collected and fixed 4 days post treatment and analyzed using multiplexed immunohistochemistry of sections stained for endothelial cells with a CD31 antibody (PECAM; Bethyl; 1:50), erythrocytes with a TER-119 antibody (BD Biosciences; 1:50), and nuclei with Hoechst 33342 (Invitrogen; 1:1000). Quantifications were made using an iCys research imaging cytometer as previously described [52].

### Statistical analysis

All experiments had an n ≥ 3 for each treatment and similar results were seen in at least two experiments. Results are expressed as means ± SEM. Statistical differences were measured by ANOVA with a Tukey posthoc test using JMP software.

## Abbreviations

VEGF: Vascular endothelial growth factor; ADSC: Adipose derived stromal cells; ECFC: Endothelial colony forming cells, HUVEC, Human umbilical vein endothelial cells; NHDF: Normal human dermal fibroblasts; EGF: Epidermal growth factor; FGF: Fibroblast growth factor; BM: Basal media; SMA: Smooth muscle actin; PDGFR-β: Platelet derived growth factor receptor beta; HGF: Hepatocyte growth factor, Bev, Bevacizumab; Ram: Ramucirumab; GSI: Gamma secretase inhibitor.

## Competing interests

BLF, DM, GFE, JS, MLS, YC, LNL, KN, MU, and SC are employees of Eli Lilly and Co. BOC, KA, and DPM are employees of Essen Biosciences.

## Authors’ contributions

BLF played a role in conception and design of the experiments, acquisition, analysis, and interpretation of the data, and writing the manuscript. BOC and KA participated in optimization of live cell imaging and in the acquisition and analysis of the data. DM, MLS, YC, LNL, KN played a role in the conception and optimization of the cord formation assay. GFE developed, optimized, and performed the in vivo vasculogenesis assays and was involved in the design and acquisition of the data. JS was involved in the acquisition, analysis and interpretation of the in vivo vasculogenesis assay. DPM and MTU were involved in the conception and design of the experiments, analysis and interpretation of the data, and in writing the manuscript. SC played a role in conception, design and optimization of the in vitro experiments and in vivo vasculogenesis assays, analysis and interpretation of the data, and in writing the manuscript. All authors read and approved the final manuscript.

## Supplementary Material

Additional file 1: Figure S1 Role of HGF in basal cords CD31 stained basal cords at 3 days treated with 10 μg/mL hIgG or anti-HGF antibody. Click here for file
