# Supplementary figures and images for: Development and characterization of a high-throughput in vitro cord formation model insensitive to VEGF inhibition
Source: J Hematol Oncol. 2013 Apr 27;6:31. doi: 10.1186/1756-8722-6-31 (PMC3648446; doi:10.1186/1756-8722-6-31)

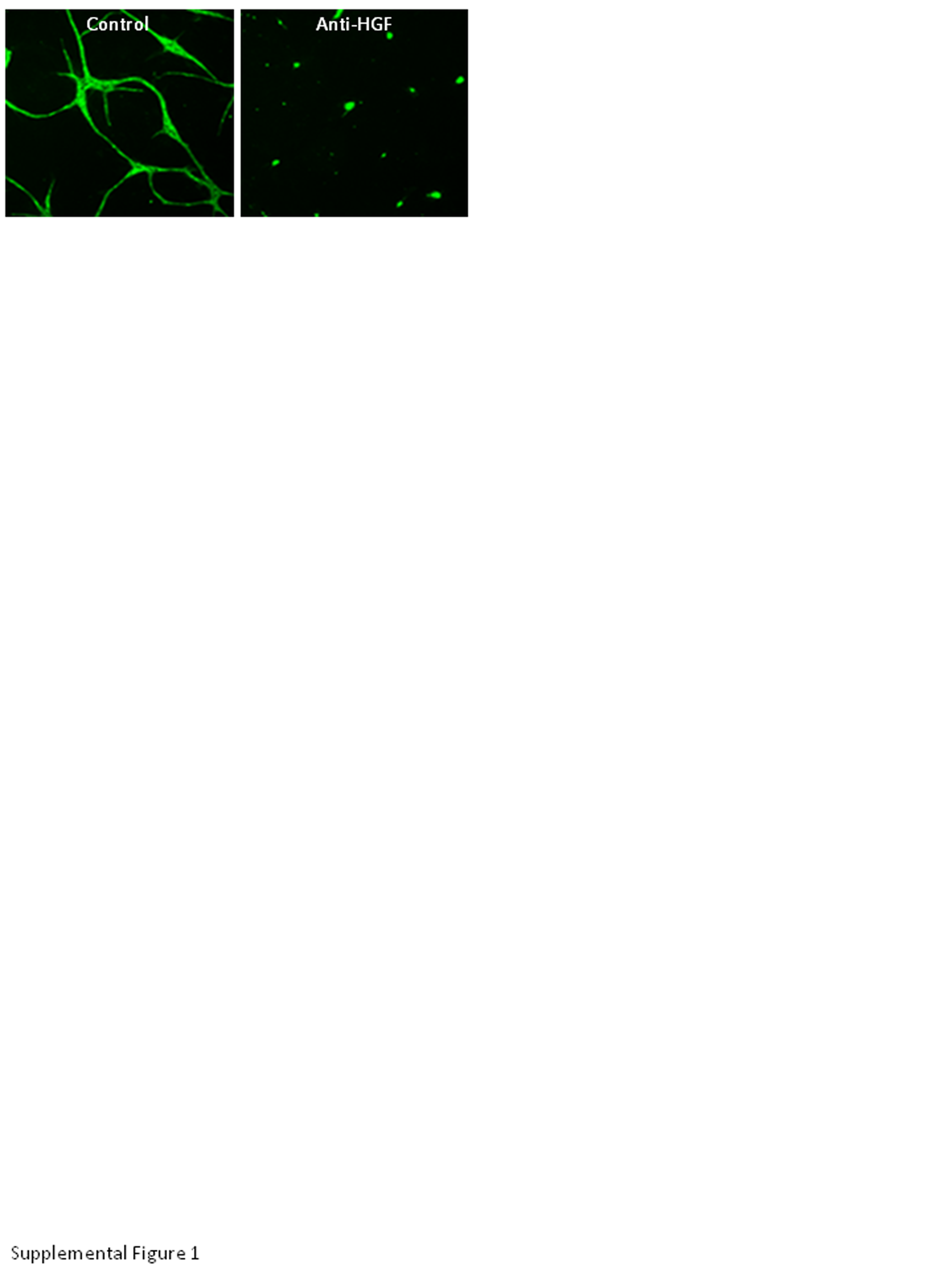

Supplement: Additional file 1: Figure S1 — Role of HGF in basal cords CD31 stained basal cords at 3 days treated with 10 μg/mL hIgG or anti-HGF antibody. [file 1756-8722-6-31-S1.tiff]
